# Somatic mutations in Brazilian patients with paroxysmal nocturnal hemoglobinuria: a comprehensive analysis

**DOI:** 10.3389/fmed.2025.1472186

**Published:** 2025-02-24

**Authors:** Patricia Eiko Yamakawa, Caio Perez Gomes, Agatha Ribeiro Mendes, Caio Cesar Justino de Oliveira, Florencio Porto Freitas, Fabiana Bettoni, Ernande Xavier dos Santos, Vinicius Campos de Molla, Matheus Vescovi Gonçalves, Jessica Branquinho, Beatriz Ribeiro Nogueira, Joao Bosco Pesquero, Celso Arrais-Rodrigues

**Affiliations:** ^1^Department of Hematology, Universidade Federal de São Paulo/Escola Paulista de Medicina, São Paulo, Brazil; ^2^Department of Biophysics, Universidade Federal de São Paulo, São Paulo, Brazil; ^3^Rudolf Virchow Zentrum (RVZ), Center for Integrative and Translational Bioimaging, University of Würzburg, Würzburg, Germany; ^4^Molecular Oncology Center, Hospital Sirio Libanes, São Paulo, Brazil

**Keywords:** paroxysmal nocturnal hemoglobinuria, *PIG-A* mutation, hemolytic anemia, *PIG-A* gene, pathogenicity

## Abstract

**Background:**

Paroxysmal nocturnal hemoglobinuria (PNH) is a rare clonal hematopoietic stem cell disease characterized by acquired abnormalities in the phosphatidylinositol glycan class A (*PIG-A*) gene.

**Methods:**

This study analyzed *PIG-A* gene using polymerase chain reaction (PCR) followed by Sanger sequencing of 31 Brazilian patients with PNH, including 23 with classical PNH and 8 with subclinical PNH (aplastic anemia and a PNH clone).

**Results:**

A diverse spectrum of acquired *PIG-A* variants was identified, encompassing insertions, deletions, and single-base substitutions. The majority of variants identified (17 out of 29) were deemed likely pathogenic for paroxysmal nocturnal hemoglobinuria (PNH). Six variants have undetermined significance (VUS) and six variants are probably benign. Somatic variants exhibited variability in type and location among the patients, with a predominance of small deletions and simple base changes. Notably, 41% of the variants were frameshift and 35% were missense. Among the 23 patients with hemolytic PNH, 19 had at least one detectable pathogenic variant. Subclinical PNH cases were characterized solely by polymorphisms.

**Conclusion:**

In conclusion, the somatic variants in Brazilian PNH patients displayed variability in both site distribution and type. Contrary to mutational hotspots observed in previous studies, none were identified in this cohort. No specific correlation between the clinical characteristics of hemolytic PNH patients and their variants was found, likely due to the extensive variety of mutations.

## Introduction

Paroxysmal nocturnal hemoglobinuria (PNH) is a rare clonal hematopoietic stem cell disease characterized by a wide spectrum of clinical manifestations, including intravascular hemolysis, a hypercoagulable state, and bone marrow failure. Its global incidence is estimated in 1–1.5 cases per million individuals per year (1).

PNH pathogenesis involves the non-malignant clonal expansion of hematopoietic stem cells with acquired abnormalities in the phosphatidylinositol glycan class A (*PIG-A*) gene. *PIG-A* is an X-linked gene that encodes a protein required for glycosylphosphatidylinositol (GPI) anchor synthesis (2). The *PIG-A* gene comprises six exons and five introns spanning 17 kb (3). Exon 1 (23 bp) is a non-coding 5′-untranslated region, while exon 2 (777 bp) contains half of the coding region. Exons 3 (133 bp), 4 (133 bp), and 5 (207 bp) contain portions of the coding region, and exon 6 (2,316 bp) encompasses the remaining coding region and the 3′-untranslated region. The 5′-flanking region (583 bp) has promoter activity. *PIG-A* is located on the X chromosome’s short arm (Xp22.1) (2). A single inactivating mutation can produce a PNH phenotype since men have only one X chromosome, and women exhibit X chromosome inactivation (lyonization) in somatic cells, including hematopoietic stem cells (4).

Somatic *PIG-A* pathogenic variants in hematopoietic stem cells result in partial or complete absence of GPI-linked proteins normally present on the cell membrane. CD55 (decay-accelerating factor) and CD59 (membrane inhibitor of reactive lysis) are essential complement regulatory proteins anchored via GPI. In PNH patients, CD55 and CD59 deficiencies in the clonal blood cells lead to increased red blood cell susceptibility to complement system-mediated cell activaction, cell damage and hemolysis (5, 6).

Clinical observations highlight the heterogeneous nature of PNH patients, with variations in symptom manifestations, hemolysis intensity, and responses to eculizumab treatment. Some patients persistently experience hemolysis, including an extravascular component. A wide array of acquired *PIG-A* variants has been documented (7), predominantly featuring frameshift mutations (8, 9). Interestingly, these variants do not correlate with clinical factors such as anemia severity or thrombosis presence in PNH patients. This study aims to characterize the mutational profile of 31 Brazilian patients with PNH clones.

## Methods

Patients harboring PNH clones, identified by flow cytometry, were selected for this study. Flow cytometry analysis was performed using monoclonal antibodies targeting GPI-linked surface antigens like CD59, CD157, and FLAER, as recommended by Borowitz et al. (10). Detection of at least 0.01% of cells lacking GPI-linked marker expression was considered a positive result. The local research ethics committee approved the study, and all patient samples and clinical information were collected following written informed consent.

The patients were classified in three groups according to International PNH Interest Group (11): group 1, classic PNH, characterized by large PNH clones and episodes of intravascular hemolysis; group 2, PNH associated with another hematologic disease, characterized by clinical/laboratory presence of hemolysis in patients who also have another concomitant diagnosis, such as aplastic anemia; and group 3, subclinical PNH, characterized by patients who have small PNH clones without clinical or laboratory manifestations of hemolysis.

### Sample collection and DNA extraction

Peripheral blood samples (10 mL) were collected in EDTA-containing tubes and processed as previously described (12).

Briefly, samples underwent centrifugation to separate plasma from peripheral blood cells, followed by isolation of white blood cells (buffy coat) stored at −80°C until use.

DNA extraction from the buffy coats was performed using the QIAamp DNA Blood Mini QIAcube Kit (Qiagen, Valencia, CA, USA) on the QIAcube Connect automated nucleic acid extractor (Qiagen, Courtaboeuf, France).

### Sanger sequencing

Primers for *PIG-A* sequencing were designed to anneal to intronic sites flanking the exonic regions, as previously described (2) (see Table 1). Polymerase chain reaction (PCR) assays utilized 25 μL reaction volumes containing genomic DNA (50 ng), 1 × Taq DNA polymerase buffer (Invitrogen, Thermo Fisher Scientific, Waltham, MA, USA), 1.50 or 1.25 mM MgCl2 (see Table 1), 0.2 mM dNTPs (GE Healthcare, Little Chalfont, UK), 1 U of Taq DNA polymerase (Invitrogen), and 0.16 μM of each primer. The PCR conditions included an initial denaturation at 94°C for 5 min, followed by 35 cycles of 30 s at 94°C, 30 s at 55 or 59°C (see Table 1), and 1 min at 72°C, concluding with a final extension at 72°C for 6 min. The resulting PCR products were analyzed on 1% agarose gel and subsequently sequenced using the BigDye™ Terminator v3.1 Cycle Sequencing Kit (Applied Biosystems, Thermo Fisher Scientific) on an ABI 3130xl Genetic Analyzer (Applied Biosystems).

**Table 1:** PCR primers and conditions.

Segments	Primer forward*	Primer reverse*	Covered region (GRCh38/hg38 Assembly)	Covered Exon	Amplified product (bp)	Annealing temperature (°C)
*I*	*5′-cagacctcgtatgtttccctcag-3′*	*5′-agaacagccccatccgaaagcg-3′*	*chrX:15335439-15336103*	*1*	*665*	*55*
*II*	*5′-gagctgagatcctgttttactct-3′*	*5′-gaaaaagaactatgtgaatggat-3′*	*chrX:15331536-15332022*	*2*	*487*	*55*
*III*	5′-tcgaaaaggcatccgttac-3′ **	*5′-gccaaacaatcattatatacaag-3′*	*chrX:15331131-15331703*	*2*	*573*	*55*
*IV*	*5′-tggattctcagtcgttctggtga-3′*	*5′-cttctccctcaagacaacatgaa-3′*	*chrX:15325879-15326122*	*3*	*244*	*55*
*V*	*5′-tcactcctttcttcccctctc-3′*	*5′-gtacgtgaaacatcaagtaagag-3′*	*chrX:15324608-15325187*	*4 and 5*	*580*	*55*
*VI*	*5′-ggtcattgtttatcatgggacag-3′*	*5′-tcttacaatctaggcttccttc-3′*	*chrX:15321485-15321845*	*6*	*361*	*59*

* primer sequences derived from Iida et al. (2).

** primer designed in-house.

### Mutation pathogenicity prediction and allelic frequency assessment

To evaluate the identified variants, we employed databases and in silico pathogenicity prediction tools in line with established literature and the parameters proposed by the American College of Medical Genetics and Genomics (ACMG) (13).

ClinVar-dbSNP (14): A public archive maintained by the National Institutes of Health that provides information on human genetic variants and their association with diseases.

REVEL (15): A tool that integrates 18 pathogenic prediction scores from 13 different tools, focusing on rare pathogenic variants. Scores above 0.5 indicate a higher likelihood of deleteriousness, with recent studies suggesting thresholds above 0.8 for deleterious variants and below 0.4 for benign ones.

PolyPhen (16): A tool that predicts the probability of a mutation being damaging by analyzing the impact of the altered amino acid on protein function.

MutationTaster (17): A tool capable of analyzing intronic, synonymous, and short indel mutations, predicting their effects. It incorporates data from sources such as 1,000 Genomes, ClinVar, and gnomAD to assess variant pathogenicity.

CADD (18): A tool that scores single nucleotide variants and small insertions or deletions based on their molecular functionality and pathogenicity, providing a probability score for deleterious effects.

gnomAD (19): A comprehensive database containing data from 76,156 whole-genome sequences and 125,748 exome sequences, used for sequence analysis.

Allelic frequencies were assessed using the 1,000 Genomes Project database, which includes over 88 million variants, covering more than 99% of SNPs with a frequency greater than 1% across diverse ancestries (20).

## Results

A total of 31 patients were included in this study. The median age was 36 years (range 19–72 years), with 55% being male. Twenty patients had classical PNH with large PNH clones (Group 1), three had classical PNH with another hematologic disease (Group 2), and eight had aplastic anemia with small PNH clones (Group 3). The clinical characteristics and laboratory features of the patients are summarized in Tables 2, 3. Among these, 16 (52%) from Groups 1 and 2 experienced hemoglobinuria, and 11 (35%) reported abdominal pain. Twenty-five patients (81%) required transfusion. Eight patients (26%) experienced thrombotic events; seven venous (22%) and one arterial (3%), Including five intra-abdominal venous thromboses (16%), with two cases (6%) being Budd-Chiari syndrome. The median hemoglobin level at sample collection was 9.1 g/dL (range 4.7–14.4 g/dL). The median lactate dehydrogenase (LDH) level was 1,455 U/L (range 159–3,989 U/L, upper limit 480 U/L). Median PNH clone sizes were 79% in monocytes (range 0.03–99.9%), 70% in granulocytes (range 0.01–99.9%), and 37% in red blood cells (range 0.01–95%).

**Table 2:** Patient and disease characteristics.

Characteristics	
Number of patients	31
Age at diagnosis, years	
Median (Range)	36 (19–72)
Male, no. (%)	17 (55)
Diagnosis, no. (%)	
Classical PNH	20 (65)
PNH associated with other hematologic disease	3 (9)
Aplastic anemia with PNH clone	8 (26)
Hemoglobinuria, no. (%)	16 (52)
Abdominal pain, no. (%)	11 (36)
Dysphagia, no. (%)	5 (16)
Fatigue, no. (%)	23 (74)
Headache, no. (%)	7 (23)
Venous thromboembolism, no. (%)	7 (22)
Arterial thrombosis, no. (%)	1 (3)
Transfusion required, no. (%)	25 (80)
Eculizumab treatment, no. (%)	19 (61)
Hemoglobin, g/dL	
Median (Range)	9.1 (4.7–14.4)
Leucocytes, 10^9^/L	
Median (Range)	4.07 (1.51–9.44)
Platelets, 10^9^/L	
Median (Range)	74 (8–213)
Lactate dehydrogenase, U/L	
Median (Range)	1,455 (159–3,989)
PNH erythrocyte clones	
Median (Range)	37% (0.01–95%)
PNH granulocyte clones	
Median (Range)	70% (0.01–99%)
PNH monocyte clones	
Median (Range)	79% (0.03–99%)

PNH, paroxysmal nocturnal hemoglobinuria.

**Table 3:** Clinical characteristics and *PIG-A* mutations in PNH patients.

Patient	Group	Age	Sex	Venous thrombosis	Arterial thrombosis	Local	Need of transfusion	Hemoglobinuria	Abdominal pain	Eculizumab	Hemoglobin	LDH	Granulocyte clone	Monocyte clone	*PIG-A* mutations
1	1	32	M	N	N		N	S	N	Y	6.60	2277	90.00	90.00	c.1004G>A (p.Gly335Glu)
2	1	41	F	N	N		Y	Y	N	Y	4.70	2.812	92.00	79.00	c.564T>G (p.Ser188Arg) c.-129A>G c.-427G>C
3	1	32	M	Y	N	Mesenteric	Y	N	Y	Y	9.40	1.455	70.00	100.00	c.984_986del (p.Val329del) c.982G>T (p.Val328Phe) c.-129A>G
4	1	34	F	N	N		N	Y	Y	Y	10.70	1.891	70.00	81.00	c.549T>A (p.Cys183*) c.-129A>G
5	1	25	M	N	N		Y	N	N	Y	5.90	1.603	55.00	98.00	c.264dup (p.Val89Serfs*41) c.840del (p.His281Metfs*10) c.984_986del (p.Val329del) c.-129A>G
6	1	57	F	N	N		Y	Y	N	Y	9.20	3.989	99.56	99.86	c.944G>T (p.Cys315Phe) c.-129A>G c.-427G>C
7	1	39	F	Y	N		Y	Y	Y	Y	9.19	3.720	73.81	90.50	c.878_896del (p.Asp294Phefs*7) c.-129A>G
8	1	61	F	N	N		Y	Y	N	Y	11.10	978	70.00	98.00	c.234del (p.Gly79Alafs*16) c.832del (p.Tyr278Thrfs*13) c.*7G>T c.-129A>G
9	1	27	M	N	N		Y	Y	Y	Y	9.10	2.133	84.00	86.00	c.981+8G>A c.55C>T (p.Arg19Trp) c.-220T>A c.-129A>G
10	1	30	M	N	N		Y	Y	N	Y	8.10	3.323	99.00	93.00	c.577_581del (p.Val193Lysfs*7) c.-129A>G
11	1	39	F	Y	N	Venous cerebral thrombosis, mesenteric	Y	Y	Y	Y	8.7	773	96.00	96.50	c.577_581del (p.Val193Lysfs*7)c.910_911del (p.Ile304Phefs*8)c.-129A>G c.853C>G (p.Arg285Gly)
12	1	52	M	N	N		N	N	N	Y	13.50	492	66.50	87.00	c.548G>A (p.Cys183Tyr) c.55C>T (p.Arg19Trp) c.-220T>A c.-129A>G
13	1	28	M	N	N		Y	N	N	Y	11.50	1.662	65.00	63.00	None
14	1	57	M	Y	N	Deep vein thrombosis of lower limb, splenic thrombosis	Y	Y	Y	Y	9.60	2.586	82.00	92.00	c.981+8G>A
15	1	37	M	N	N		Y	Y	Y	Y	12.20	3.783	90.00	80.00	c.1049_1053del (p.Pro350Argfs*6) c.-129A>G
16	3	51	M	N	N		Y	N	N	N	7.20	533	0.14	0.13	c.-129A>G
17	3	27	M	N	N		Y	N	N	N	7.00	159	0.03	0.05	None
18	3	22	F	N	N		N	N	N	N	14.40	516	6.90	10.70	c.-129A>G c.-427G>C
19	3	57	F	N	N		Y	N	N	N	9.10	212	0.00	0.03	c.-129A>G
20	2	72	M	N	N		Y	N	N	N	7.80	342	19.60	21.00	None
21	2	36	M	N	N		Y	N	N	N	7.80	304	15.00	29.60	c.715+1G>A c.55C>T (p.Arg19Trp) c.-220T>A c.-129A>G
22	3	25	F	N	N		Y	N	N	N	12.00	372	4.60	3.40	c.-129A>G
23	3	20	M	N	N		N	N	N	N	13.10	326	1.50	1.80	c.-129A>G c.-427G>C
24	1	40	M	Y	Y	Budd-Chiari, stroke	Y	v	Y	N	9.7	1836	89.00	15.00	c.142G>T (p.Gly48Cys)
25	1	50	F	N	Y	Transient ischemic attack	Y	Y	N	Y	8.50	1.760	79.00	88.00	c.453del (p.Phe151Leufs*21) c.-129A>G c.-427G>C
26	1	68	F	Y	Y	Budd-Chiari, stroke	Y	Y	Y	Y	6.60	2.504	56.00	88.00	c.-129A>G c.-427G>C
27	2	64	F	N	N		S	Y	N	Y	7.8	520	36.00	52.00	c.336del (p.Leu112Phefs*13)
28	1	30	M	Y	N	Deep vein thrombosis in lower limbs	Y	N	Y	N	7.6	2.078	70.00	49.00	None
29	1	36	M	N	N		Y	Y	Y	Y	13.60	407	95.00	99.00	c.944G>A p.Cys315Tyr
30	3	19	F	N	N		Y	N	N	N	8.60	413	3.40	7.90	c.-129A>G c.-427G>C
31	3	33	F	N	N		N	N	N	N	12.20	408	5.90	6.60	c.-129A>G c.-427G>C

We detected 29 distinct variants in 27 of the 31 patients (refer to Table 4). One mutation had been previously identified: c.55C > T (p.Arg19Trp), a benign polymorphism in exon 2 already reported (Nafa et al., Endo et al.). A c.*7G > T mutation found in the 1,000 Genomes project and two SNPs, c.—427G > C and c.-129A > G, were observed in the general population and are cataloged in the 1,000 Genomes databases. Twenty-five newly identified variants had no prior descriptions in the literature.

**Table 4:** Mutations detected in this study.

Location	Variant	Type	Clinical significance		Pathogenicity prediction			Allele frequency (%)			Reference (PMID)
			ClinVar - dbSNP	ACMG Classification (Franklin)	Revel	PolyPhen	MutationTaster	CADD RAW	gnomAD	1000 genomes	
5’UTR	c.-427G > C		Benign rs6632348	Benign (BA1, BS2, BP7, BP6)	-	-	-	-	34.87	36.03	Not described
5’UTR	c.-220 T > A		Likely benign rs116740080	Likely benign (BS2, BP7, BP6)	-	-	-	-	3.06	2.76	Not described
5’UTR	c.-129A > G		- rs4830938	Benign (BA1, BP7)	-	-	-	-	75.86	86.57	Not described
Exon 2	c.55C > T (p.Arg19Trp)	Missense	Benign rs34422225	Benign (PM5, BS2, BP4, BP6)	0.059	Benign	Polymorphism automatic	0.7769	3.01	2.46	Nafa et al. (8), Nafa et al. (9), Endo et al. (11)
Exon 2	c.142G > T (p.Gly48Cys)	Missense	-	Likely pathogenic (PP3, PM2, PM5)	0.969	Probably damaging	Disease causing	3.7970	Not found	Not found	Not described
Exon 2	c.234del (p.Gly79Alafs*16)	Frameshift	-	Likely pathogenic (PVS1, PM2)	-	-	-	-	Not found	Not found	Not described
Exon 2	c.264dup (p.Val89Serfs*41)	Frameshift	-	Likely pathogenic (PVS1, PM2)	-	-	-	-	Not found	Not found	Not described
Exon 2	c.336del (p.Leu112Phefs*13)	Frameshift	-	Likely pathogenic (PVS1, PM2)	-	-	-	-	Not found	Not found	Not described
Exon 2	c.453del (p.Phe151Leufs*21)	Frameshift	-	Likely Pathogenic (PVS1, PM2)	-	-	-	-	Not found	Not found	Not described
Exon 2	c.548G > A (p.Cys183Tyr)	Missense	-	VUS (PM2, PP3, PM1)	0.894	Probably damaging	Disease causing	3.7039	Not found	Not found	Not described
Exon 2	c.549 T > A (p.Cys183*)	Nonsense	-	Likely pathogenic (PVS1, PM2)	-	-	Disease causing	5.9144	Not found	Not found	Not described
Exon 2	c.564 T > G (p.Ser188Arg)	Missense	-	VUS (PM2, PM1)	0.595	Probably damaging	Disease causing	2.9971	Not found	Not found	Not described
Exon 2	c.577_581del (p.Val193Lysfs*7)	Frameshift	-	Likely pathogenic (PVS1, PM2)	-	-	-	-	Not found	Not found	Not described
Intron 2	c.715 + 1G > A	5′-splice site	-	Likely Pathogenic (PVS1, PM2)	-	-	-	4.8141	Not found	Not found	Not described
Exon 3	c.832del (p.Tyr278Thrfs*13)	Frameshift	-	Likely pathogenic (PVS1, PM2)	-	-	-	-	Not found	Not found	Not described
Exon 3	c.840del (p.His281Metfs*10)	Frameshift	-	Likely Pathogenic (PVS1, PM2)	-	-	-	-	Not found	Not found	Not described
Exon 4	c.853C > G (p.Arg285Gly)	Missense	-	VUS (PM2)	0.484	Benign	Disease causing	4.1092	Not found	Not found	Not described
Exon 4	c.878_896del (p.Asp294Phefs*7)	Frameshift	-	Likely pathogenic (PVS1, PM2)	-	-	-	-	Not found	Not found	Not described
Exon 4	c.910_911del (p.Ile304Phefs*8)	Frameshift	-	Likely pathogenic (PVS1, PM2)	-	-	-	-	Not found	Not found	Not described
Exon 4	c.944G > T (p.Cys315Phe)	Missense	-	VUS (PM2, PP3)	0.809	Probably damaging	Disease causing	3.2341	Not found	Not found	Not described
Exon 4	c.944G > A p.Cys315Tyr	Missense	-	VUS (PM2, PP3)	0.778	Possibly damaging	Disease causing	3.1584	Not found	Not found	Not described
Intron 4	c.981 + 8G > A	Missense	Benign rs778602062	Benign (BS1, BS2, BP4, BP6)	-	-	-	0.1793	0.03	2.46	Not described
Exon 5	c.981 + 2 T > C	5′-splice site	-	Likely pathogenic (PVS1, PM2)	-	-	-	4.6612	Not found	Not found	Not described
Exon 5	c.982G > T (p.Val328Phe)	Missense	-	VUS (PM2, PP3)	0.846	Probably damaging	Disease causing	3.9554	Not found	Not found	Not described
Exon 5	c.984_986del (p.Val329del)	Frameshift	-	Likely pathogenic (PM4, PM5, PM2)	-	-	-	-	Not found	Not found	Not described
Exon 5	c.984del (p.Val329*)	Frameshift	-	Likely Pathogenic (PVS1, PM2)	-	-	-	-	Not found	Not found	Not described
Exon 5	c.1004G > A (p.Gly335Glu)	Missense	-	Likely pathogenic (PP3, PM2)	0.939	Probably damaging	Disease causing	3.7012	Not found	Not found	Not described
Exon 5	c.1049_1053del (p.Pro350Argfs*6)	Frameshift	-	Likely Pathogenic (PVS1, PM2)	-	-	-	-	Not found	Not found	Not described
3’-UTR	c.*7G > T		Benign rs142178534	Benign (BS2, BP7, BP6)	-	-	-	0.3066	0.13	0.37	Not described

Predominantly, point mutations were observed, including 16 variants involving a simple exchange of base pairs, one insertion, 10 small deletion mutations (ranging from one to five base pairs), and one large deletion (19 base pairs). The alterations were categorized as follows: 12 frameshift (41%), 10 missense (35%), two splice site (7%), and one nonsense variant (3%). Four mutations (14%) were found in non-coding regions.

**Figure 1:**
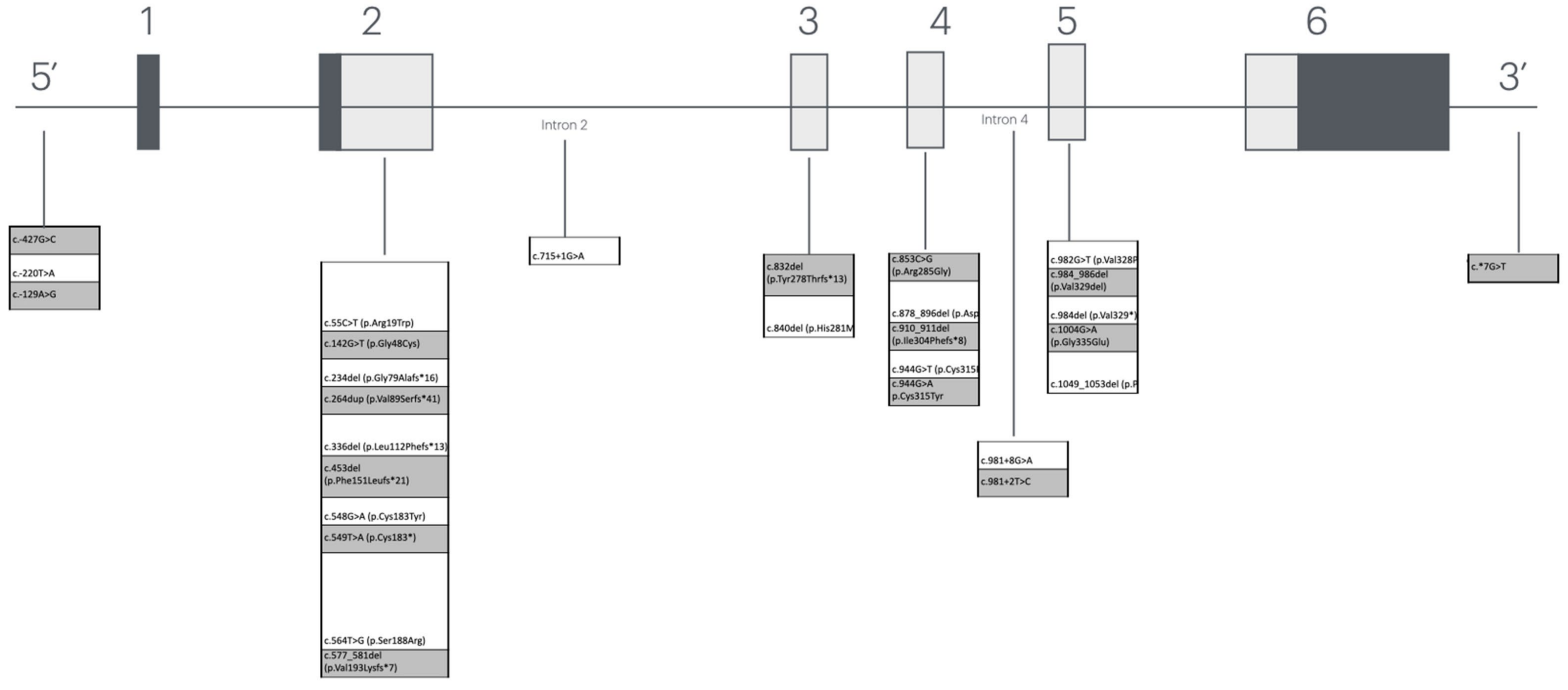
Representation of the location of mutations found in the PIG-A gene.

Variants in the *PIG-A* gene were distributed throughout the gene: three in the 5′ region, 10 in exon 2, two in exon 3, five in exon 4, five in exon 5, one in intron 2, two in intron 4, and one in the 3′ region (Figure 1).

Four patients showed no detectable variants: two with hemolytic PNH (group 1), one with aplastic anemia and PNH clone (group 2) and one with subclinical PNH (group 3).

### In silico analysis

The impact of variants found in these patients was assessed using software tools outlined in the methods section. The majority of variants identified (17 out of 29) were deemed likely pathogenic for paroxysmal nocturnal hemoglobinuria (PNH).

Six variants have undetermined significance (VUS). c.548G > A, c 564 T > G, c.944G > T, c.944G > A, and c.982G > T were predicted as probably pathogenic by PolyPhen and Mutation taster, and have Revel scores above 0.5, indicating a higher likelihood of deleteriousness. Inconsistent scoring was noted for the c.853C > G (p.Arg285Gly) variant; MutationTaster rated it as likely pathogenic, whereas PolyPhen-2 assessed it as benign.

Intronic variants were evaluated. The c.981 + 8G > A mutation was found to have no impact on splicing, while c.715 + 1G > A and c.981 + 2 T > C were predicted to potentially affect splicing, suggesting they may have pathological consequences.

Two variants, c.*7G > T and c.55C > T, were categorized as benign, with the latter also described as a polymorphism in multiple studies.

Three variants in the 5′ regulatory region—c.-220 T > A and two polymorphisms, c.-427G > C and c.-129A > G (both with high frequencies in the general population as described in Table 4) — were classified as having undetermined effects, suggesting they may exhibit benign behavior.

Out of the 23 patients with hemolytic PNH, 19 presented with at least one variant assessed as pathogenic. Notably, Patient 9, despite harboring variants generally regarded as benign or of unknown significance, such as c.55C > T (p.Arg19Trp), c.-129A > G, c.-220 T > A, and a c.981 + 8G > A intronic variant (which according to ACMG classification does not affect splicing), displayed classical PNH with significant hemolysis and a large PNH clone presence (99%). Similarly, Patient 26, diagnosed with classical PNH, had only the SNPs c.-427G > C and c.-129A > G in the 5′ regulatory region, which are not known to have clinical impact. In addition, two patients with hemolytic PNH did not show any variant in the *PIG-A* gene.

A significant proportion of patients (15 out of 23, or 65%) with hemolytic PNH possessed multiple variants concurrently, with the predominant ones being potentially pathogenic in conjunction with a polymorphism. Specifically, five patients carried more than one variant likely to be pathogenic. No clear correlation was observed between the clinical presentation and the type or quantity of variants identified.

Among the variants, three were confirmed as polymorphisms. The c.55C > T (p.Arg19Trp) polymorphism was detected in three individuals within the hemolytic PNH subgroup. Two SNPs, c.-427G > C and c.-129A > G, were frequently observed; c.-129A > G was present in 22 of the 31 patients (approximately 70%), while c.-427G > C was found in eight patients (around 26%). These polymorphisms have high frequency related in population, approximately 86 and 36%, respectively, in 1000 Genomes database, and were the only genetic variants identified in seven of eight patients from Group 3, who had subclinical PNH manifestations.

## Discussion

To the best of our knowledge, this study represents the first comprehensive evaluation of PNH cases using *PIG-A* gene sequencing in Brazil. We identified 29 somatic variants in 27 out of 31 patients. Among these, only one variant was previously characterized in the literature. Three patients had the c.55C > T variant, initially described by Iida et al. (2). The majority of variants identified (17 out of 29) were deemed likely pathogenic for paroxysmal nocturnal hemoglobinuria (PNH). Six variants have undetermined significance (VUS) and six variants are probably benign.

Since Miyata et al.’s landmark discovery in 1993 (21), which linked PNH pathogenesis to variants in the X-linked *PIG-A* gene, over a hundred variants have been documented worldwide (9, 22–28). In a smaller cohort, De Carvalho et al. (29) described three Brazilian patients with classical PNH. They used conformation-sensitive gel electrophoresis followed by direct sequencing to identify three distinct variants.

The spectrum of *PIG-A* variants is broad, predominantly composed of single-nucleotide substitutions, small insertions, and deletions, with large gene deletions being relatively rare. Frameshift mutations often result in premature stop codons, potentially leading to truncated proteins with compromised or lost function. *PIG-A* variants include nonsense, splice-site variants affecting mRNA processing, and missense that result in single amino acid changes. Some missense variants may confer partial protein functionality, explaining the partial deficiency of GPI-linked proteins in the PNH type II phenotype, while others may lead to a complete loss of function. Our findings resonate with existing literature, wherein frameshift and missense variants are reported frequently; in our study, these accounted for 41 and 35%, respectively. Notably, missense variants with widespread effects are usually situated in pivotal regions; here, four out of nine missense mutations were located in exon 2, which is consistent with previous suggestions that this exon is a critical site for function-affecting variants.

Variants in the *PIG-A* gene are distributed across the coding region, occurring at random with no significant hotspots, except for a specific sequence in exon 2 reported by some studies (22). The majority of variants were identified in exon 2, likely due to its size and coding significance within the *PIG-A* gene.

In our study, 19 out of 23 patients with hemolytic PNH exhibited more than one *PIG-A* variant, suggesting a possible oligoclonal nature of the disease, as previously proposed (8, 30). This theory is supported by the finding that multiple hematopoietic stem cell lineages can acquire distinct *PIG-A* variants and propagate independently. Genotypic mosaicism within T-cell clones, as demonstrated by Endo et al. (30), underlines the phenotypic diversity observed in PNH. This genotypic diversity within PNH may be due to a selective autoimmune pressure, a hypothesis not yet conclusively proven (31, 32).

Interestingly, patients with concomitant variants did not exhibit clinical differences compared to those with a single mutation. This observation reinforces the idea that PNH is a complex disorder with a multifactorial pathogenesis. In cases of subclinical PNH, no pathogenic variants were identified; only polymorphisms with a high incidence in the general population were present. This finding, coupled with the absence of a sorting method for GPI-deficient leukocytes, suggests that these polymorphisms are likely benign.

The absence of detectable *PIG-A* variants in four patients, including one with subclinical PNH and three with hemolytic PNH, suggests that mutations may be located in regions not examined by our study, such as non-coding intronic regions. It’s noteworthy that other genes involved in GPI-anchor biosynthesis, like *PIG-T* and *PIG-M*, have been implicated in GPI deficiency, but pathogenic variants in these genes are rare and typically associated with autosomal chromosomes, requiring biallelic mutations to manifest (33, 34). These cases often present with a broader clinical spectrum, including developmental delay and epilepsy, which differs from the hemolysis predominant in PNH (35).

The relationship between aplastic anemia and PNH clones, as well as the role of immune selection, provides an interesting angle on the survival advantage of PNH clones (36, 37). This is highlighted by the varying mutation profiles in patients with aplastic anemia and myelodysplastic syndrome compared to those with classical PNH, as reported by Okamoto et al. (38). The diversity of *PIG-A* variants and their association with different disease phenotypes underscore the complexity of PNH pathogenesis.

Furthermore, the detection of somatic *PIG-A* variants in individuals with normal hematopoiesis and their prevalence in healthy individuals indicate that these mutations alone are not sufficient for clonal expansion (39–41). The existence of additional genetic abnormalities in PNH patients, such as variants in genes like *TET2* and *JAK2*, suggests a multistep clonal evolution that may contribute to the expansion of PNH clones (42, 43). Additionally, the expression of *HMGA2*, an architectural transcription factor, has been found to be abnormally high in some PNH patients, potentially contributing to clonal expansion (44).

In terms of clinical manifestations, our study did not reveal a clear correlation between the mutational profile and the severity of hemolytic PNH. This may be due to the wide range of mutations observed. The study also reflects on additional genetic factors, such as *BMPR2* and *THBD*, that may contribute to the risk of thrombosis in PNH patients (45).

In a comprehensive analysis by Chen et al. (46), the clinical implications of variants were elucidated through whole-exome sequencing of genes commonly mutated in PNH, including *PIG-A*, *BCORL1, RUNX1T1, MAP3K4, CSMD1, NOTCH1, FANCD2, PEG3, DIS3*, and *SETBP1*. They found correlations between specific variants and clinical features: the *RUNX1T1* mutation was linked to larger PNH clones, higher levels of unconjugated bilirubin, and lower hemoglobin levels; the *BCORL1* mutation tended to occur in younger patients; the *SRRD* mutation was tied to visceral thrombosis; and the *EGR4* mutation was associated with myocardial infarction. This emphasizes the potential for targeted mutation analysis to predict clinical outcomes and tailor prophylaxis in PNH patients.

To summarize, the genetic landscape of PNH in Brazilian patients is diverse, with a variety of variant sites and types, and no significant mutational hotspots have been identified. The variants observed were predominantly small deletions and single-nucleotide alterations. Pathogenicity prediction tools suggested that a significant proportion of these variants might play a pathogenic role in the disease: 17 variants were likely pathogenic and five of the six VUS described were probably damaging according to the tools of pathogenicity prediction. In the cohort with hemolytic PNH, a majority (19 out of 23) had variants deemed pathogenic. Interestingly, only polymorphisms, which are common in the general population and likely benign, were detected in patients with subclinical PNH.

The study did not establish a direct correlation between the clinical presentation of hemolytic PNH and specific variants, which may be due to the heterogeneity of the genetic alterations observed. This finding suggests that PNH severity and clinical manifestations are influenced by a complex interplay of factors beyond the *PIG-A* gene variants alone.

To enhance our understanding of PNH’s heterogeneity, further research involving larger cohorts, including patients from different geographical regions, and a broader analysis of genetic variants, is essential. Such investigations may uncover additional variants implicated in PNH, offering insights into the pathogenesis of the disease and potentially informing the development of personalized therapeutic approaches.

## Data Availability

The datasets presented in this study can be found in online repositories. The names of the repository/repositories and accession number(s) can be found in the article/supplementary material.
